# Overexpression of *MePMEI1* in Arabidopsis enhances Pb tolerance

**DOI:** 10.3389/fpls.2022.996981

**Published:** 2022-09-16

**Authors:** Yangjiao Zhou, Ruimei Li, Shijia Wang, Zhongping Ding, Qin Zhou, Jiao Liu, Yajia Wang, Yuan Yao, Xinwen Hu, Jianchun Guo

**Affiliations:** ^1^School of Life Sciences, Hainan University, Haikou, China; ^2^Key Laboratory of Biology and Genetic Resources of Tropical Crops, Institute of Tropical Bioscience and Biotechnology, Chinese Academy of Tropical Agricultural Sciences, Haikou, China; ^3^Key Laboratory for Biology and Genetic Resources of Tropical Crops of Hainan Province, Hainan Institute for Tropical Agricultural Resources, Haikou, China

**Keywords:** plant resistance, Pb, PMEI, cell wall, cassava

## Abstract

Pb is one of the most ubiquitously distributed heavy metal pollutants in soils and has serious negative effects on plant growth, food safety, and public health. Pectin methylesterase inhibitors (PMEIs) play a pivotal role in regulating the integrity of plant cell walls; however, the molecular basis by which PMEIs promote plant resistance to abiotic stress remains poorly understood. In this study, we identified a novel PMEI gene, *MePMEI1*, from *Manihot esculenta*, and determined its role in plant resistance to Pb stress. The expression of *MePMEI1* was remarkably upregulated in the roots, stems, and leaves of cassava plants following exposure to Pb stress. An analysis of subcellular localization revealed that the MePMEI1 protein was localized in the cell wall. MePMEI1 inhibited commercial orange peel pectin methyltransferase (PME), and the expression of *MePMEI1* in Arabidopsis decreased the PME activity, indicating that MePMEI1 can inhibit PME activity in the cell wall. Additionally, the overexpression of *MePMEI1* in Arabidopsis reduced oxidative damage and induced the thickening of cell walls, thus contributing to Pb tolerance. Altogether, the study reports a novel mechanism by which the *MePMEI1* gene, which encodes the PMEI protein in cassava, plays an essential role in promoting tolerance to Pb toxicity by regulating the thickness of cell walls. These results provide a theoretical basis for the *MePMEI1*-mediated plant breeding for increasing heavy metal tolerance and provide insights into controlling Pb pollution in soils through phytoremediation in future studies.

## Introduction

The cell walls of plants comprise polysaccharides, proteins, and polymers. They are the first barrier against external damage and play important roles in plant stress resistance. Pectin is the most complex polysaccharide in higher plants and is particularly rich in galacturonic acid (GalA). GalA consists of rhamnogalacturonan II (RGII), rhamnogalacturonan I (RGI), apiogalacturonan (AGA), xylogalacturonan (XGA), and homogalacturonan (HG) units, which are differentiated based on the diversity of their side chains and backbone structures (Atmodjo et al., 2013; Wormit and Usadel, 2018). Approximately 65% of pectin consists of HG, which is a linear polymer composed of α-1,4-D-galacruronic acid (α-D-GalA) units (Atmodjo et al., 2013). Highly methylesterified HG (~80%) is secreted into the cell wall of plants following synthesis in the Golgi apparatus and is subsequently modified by a variety of pectinases. In particular, pectin methylesterases (PMEs) catalyze the de-methylesterification of HG in plant cell walls, which results in the release of protons and methanol. HG is de-methylesterified by PMEs *via* two different modes, as described hereafter. The block-wise de-methylesterification of HG promotes the biochemical characteristics of plant cell walls *via* interactions with Ca^2+^. In random de-methylesterification, HG acts as a substrate for degradation by a series of enzymes, including pectate lyase, which reduces the firmness of cell walls (Pelloux et al., 2007; Wolf et al., 2009). Pectin methylesterase inhibitors (PMEIs) interact with pectin methyltransferases (PMEs) to regulate the activities of the latter by occupying their pectin binding site (Jolie et al., 2010; Lionetti, 2015). Therefore, PME-PMEI complexes have an essential role in regulating the degree of methylesterification of HG, which in turn influences the properties of pectin, maintenance of cellular morphology, and stability of the intercellular layer (Di Matteo et al., 2005) and involves plant stress resistance.

PMEIs belong to the large PF4043 protein superfamily, which comprises diverse family numbers that have been widely identified in various plants, including *Actinidia deliciosa, Linum usitatissimum, Oryza sativa*, and others. Numerous studies investigating the biological functions of PMEIs reported that PMEIs are involved in diverse developmental processes, including the growth of pollen tubes (Zhang et al., 2010), morphogenesis of plant cells (Haas et al., 2020; Lin et al., 2022), growth of hypocotyl and roots (Sénéchal et al., 2015; Hocq et al., 2016), fruit development and ripening (Reca et al., 2012; Lionetti et al., 2015), and seed mucilage extrusion (Kunieda et al., 2013). Additionally, numerous studies demonstrated that PMEIs participate in plant resistance to biotic and abiotic stresses (Coculo and Lionetti, 2022). For instance, the overexpression of PMEIs in wheat, pepper, and tobacco reduces the susceptibility to fungal, bacterial, and viral infections, thus increasing resistance to pathogens (Lionetti et al., 2007, 2014; Lionetti, 2015; Del Corpo et al., 2020). In particular, the overexpression of the *CaPMEI1* gene of *Capsicum annuum*, the *OsPMEI* gene of *Oryza sativa*, the *CbPMEI1* gene of *Corydalis bungeana*, and the *AtPMEI13* gene of *Arabidopsis thaliana* in the model plant Arabidopsis is associated with enhanced tolerance to drought, salt, heat, and other abiotic stresses (An et al., 2008; Nguyen et al., 2016; Chen et al., 2018). These findings demonstrate that PMEIs are responsible for conferring resistance to plants against stressors arising due to the increasing environmental changes, including global climate change, environmental pollution, and others, resulting from anthropogenic activities.

Heavy metals are the main pollutants in the environment and cause irreversible damages to living organisms. Pb is a ubiquitously distributed heavy metal pollutant in soils and is regarded as a non-essential element for plant growth. In particular, excessive exposure to Pb has negative effects on the development, morphology, photosynthetic processes, and yield of plants (Ma et al., 2022). Furthermore, some studies on pepper, quinoa, and rose suggest that the effects of Pb stress on plant growth are possibly attributed to the reduction in water content, dry weight, and other essential biochemical substances, including dehydroascorbate, monodehydsroascorbate, and photosynthetic pigments (Kaya, 2021; Amjad et al., 2022). In contrast, Pb exposure or accumulation increases the activity of antioxidant enzymes in various plants, indicating the potential role of antioxidants in plant resistance to Pb stress (Aboelkassem et al., 2022; Akay, 2022). Numerous studies reported that the metal transporter genes, *AtACBP1, AtCNGCs*, and *AtHMA3*, in Arabidopsis are associated with the transportation and absorption of heavy metals such as Pb (Morel et al., 2008; Du et al., 2015; Moon et al., 2019). A study by Zhang et al. (2020) additionally demonstrated that the *FLS1* gene regulates the accumulation of flavonols and plays a crucial role in plant resistance to Pb stress (Zhang et al., 2020). However, the precise mechanism underlying resistance to Pb stress remains poorly understood.

Cassava (*Manihot esculenta* Crantz) is a significant food and bioenergy crop that is distributed in subtropical and tropical regions worldwide. Ethanol is the major economical fuel of industrial plants, which makes cassava a potential substitute for gasoline. Consequently, the technologies used for breeding cassava plants have gained increasing attention (Dai et al., 2006; Li et al., 2017; Otun et al., 2022). Some studies investigated the effects of various environmental stressors, including drought, salt, and cold on cassava plants (Hu et al., 2015; Ou et al., 2018; Chang et al., 2019). It has been reported that cassava can grow well in soils that are contaminated with heavy metals and can improve soil fertility and acidity for soil reclamation (Shili et al., 2020). It has been demonstrated that *MeGLYI-13* improves resistance to Fe toxicity stress in Arabidopsis and yeasts (Tang et al., 2022). However, there are no reports on the mechanism underlying the resistance of cassava to Pb stress to date. As aforementioned, it is widely considered that PMEIs are involved in plant stress resistance; hence, they probably obtain the ability to confer resistance to heavy metal stress. In this study, we employed the Basic Local Alignment Search Tool (BLAST) algorithm for searching various databases and determined that the *MePMEI1* gene encoding the PMEI protein of cassava had the highest homology to *AtPMEI13* and *CbPMEI1*. The amino acid sequence similarities of MePMEI1 with the proteins encoded by *AtPMEI13* and *CbPMEI1* were 55.5 and 55.6%, respectively. Following gene identification, we determined the role of *MePMEI1* in the resistance of cassava plants to heavy metal stress, and the findings demonstrated that *MePMEI1* enhances tolerance to heavy metal (Pb) toxicity in transgenic Arabidopsis plants.

## Materials and methods

### Multiple sequence alignment

Information regarding the putative MePMEI1 protein of *M. esculenta* (Manes.06G136000), AtPMEI13 protein of *Arabidopsis thaliana* (AT5G62360), CbPMEI1 protein of *Chorispora bungeana*, and OsPMEI36 protein of *Oryza sativa* (LOC_Os10g36500) was obtained from Phytozome (https://phytozome-next.jgi.doe.gov/). The physicochemical properties of MePMEI1 were predicted using the ProtParam tool in ExPASy (https://web.expasy.org/protparam/). Multiple sequence alignment of the aforementioned proteins was performed using DNAMAN v6 software.

### Plant materials, growth conditions, and treatment with Pb(II)

The crantz.cv.M.SC8 variety of *M. esculenta* was selected from the progeny of the CMR38-120 natural F1 hybrid asexual line introduced from Thailand by the Tropical Crops Genetic Resources Institute of the Chinese Academy of Tropical Agricultural Sciences. The crantz.cv.M.SC8 variety used herein has high yield and extensive adaptability, with strong wind resistance and seedling emergence properties. SC8 cassava seedlings were grown on Murashige Skoog (MS) gar medium from the germplasm preserved in our laboratory, for ~2 months. The tender stems were cut into small segments under sterile conditions, and each segment was ~1 cm long and had two axillary buds. The segments of the stem were then inserted into a fresh MS agar medium for growth, with the axillary buds facing upward. After growing for 45 days at 26°C under 16 h/8 h light/dark conditions (white LED, 120–150 uM/m^2^/S), the cassava seedlings were subjected to Pb stress. The roots of the cassava seedlings were first rinsed with tap water, without damaging the roots as far as possible. Then, a 1 mM solution of Pb(NO_3_)_2_ was prepared, and the cassava seedlings were placed in the solution for 0, 2, 6, 12, or 24 h at room temperature. The roots, stems, and leaves of the cassava seedlings were sampled at each of these intervals and immediately frozen in liquid nitrogen and finally stored in an ultra-low temperature refrigerator at −80°C.

### RNA extraction and cDNA synthesis

The total RNA was extracted from the samples using a Plant Total RNA Isolation Kit (Foregene Biotech, Chengdu, China). The quality of the extracted RNA was evaluated by determining the concentration of the RNA and agarose gel electrophoresis. The residual DNA was removed from the extracted RNA using dsDNase, following which the cDNA was obtained using the MonScript™ RTIII Super Mix with dsDNase (Two-Step) (Monad Biotech, Wuhan, China), according to the manufacturer's instructions.

### Real-time quantitative polymerase chain reaction (RT-QPCR)

The transcription level of *MePMEI1* in the roots, stems, and leaves of cassava plants under Pb stress was analyzed using a Real-Time PCR EasyTM-SYBR Green I kit (Foregene Biotech, Chengdu, China). *Tubulin* was considered as the reference gene, and the cDNA was used as the template for RT-qPCR. The specific primers used for RT-qPCR are enlisted in Supplementary Table 1. The RT-qPCR mixture is composed of 10 of 5 μL Real PCR Easy TM Mix-SYBR (2×), 0.2 μL of forward primer (10 μM), 0.2 μL of reverse primer (10 μM), 4 μL of cDNA template, 0.2 μL of ROX Reference Dye (50×), and 0.4 μL of DNase-free ddH_2_O. The conditions of PCR were as follows: initial denaturation at 95°C for 60 s followed by 45 cycles of denaturation at 95°C for 5 s and extension at 60°C for 30 s. The dissolution curve was finally added. Three replicates were performed for each sample. The relative expression of the target gene was calculated using the 2^−ΔΔCt^ method.

### Construction of recombinant vectors

The full-length coding sequence (CDS) of *MePMEI1* without the stop codon was obtained by PCR using the Prime STAR HS (Premix), R040 (Takara Biotech, Beijing, China). The amplified products were purified and inserted into the pCAMBIA1300-35s-GFP plant binary expression vector following digestion with the *SalI* and *BamHI* restriction enzymes for constructing the pCAMBIA1300-35s-*MePMEI1*-GFP recombinant vector. The primers used for amplification are enlisted in Supplementary Table 1. The constructed recombinant vector was confirmed by Sanger sequencing.

The full-length CDS of the *MePMEI1* gene without a stop codon and signal peptide (1-28 aa) was amplified using the PrimeSTAR enzyme (Takara Biotech, Beijing, China). The amplified products were then purified and inserted into the pET-30a (+) prokaryotic expression vector following digestion with the *NdeI* and *XhoI* restriction enzymes, for constructing the PET-30a (+)-*MePMEI1* recombinant vector. The primers used for amplification are enlisted in Supplementary Table 1. The constructed recombinant vector was confirmed by Sanger sequencing.

### Analysis of subcellular localization

The pCAMBIA1300-35s-*MePMEI1*-GFP and pCAMBIA1300-35s-GFP recombinant vectors were separately transformed into *Nicotiana benthamiana* using the leaf disc method, mediated *via Agrobacterium tumefaciens*. The seeds of wild-type (WT) tobacco were sterilized with 75% ethanol for 20 min and placed on MS agar medium under 16 h/8 h light/dark conditions (white LED, 120–150 uM/m^2^/S) at 22°C for ~1 month for transformation. *A. tumefaciens* transformed with the pCAMBIA1300-35s-*MePMEI1*-GFP and pCAMBIA1300-35s-GFP recombinant vectors were cultured in the yeast extract peptone (YEP) liquid medium at 28°C with shaking at 220 rpm to an OD_600_ of 0.8. The cultures were then centrifuged at 4,000 rpm for 10 min, following which the supernatant was discarded and the bacterial cells were resuspended in a 50 mL YEP liquid medium containing 250 μM acetosyringone. The young tobacco leaves were cut into small pieces and soaked in the solution of resuspended bacteria for 10 min. The pieces of leaves were dried on sterile filter paper and cultured in an MS agar medium containing 8 mg/L hygromycin. The MS agar medium was replaced every 2 weeks, and the concentration of hygromycin was gradually increased to 20 mg/L until the positive T0 transgenic plants were obtained. All the experiments were performed under sterile conditions. Transgenic tobacco plants were further identified by leaf PCR using 2 × M5 HiPer Superluminal mix-with blue dye (Mei5bio, Beijing, China), using specific detection primers (Supplementary Table 1). The T0-positive transgenic plants were transferred to vermiculite and cultured under 16 h/8 h light/dark conditions (white LED, 120–150 uM/m^2^/S) at 22°C for obtaining the T0 plants seeds. The seeds of T0-positive transgenic tobacco were continually screened on an MS agar medium containing 20 mg/L hygromycin. The seeds were transferred to vermiculite for obtaining the seeds of T1 plants, and the seeds of transgenic T1 tobacco were germinated to obtain the T2 homozygous lines. The tips of the roots of T2 transgenic tobacco seedlings were used for determining and visualizing the localization of MePMEI1 by laser confocal microscopy.

### Expression, purification, and western blotting of recombinant protein

**Expression of recombinant protein:** The PET-30a (+)-*MePMEI1* recombinant vector was transformed into the BL21 Star (DE3) strain of *Escherichia coli*. The recombinant bacterial cells were cultured in Luria–Bertani (LB) liquid medium at 37°C with shaking at 220 rpm for 4 h to an OD_600_ of 0.6–0.8. For optimizing the induction conditions, the cultured cells were induced under different conditions, including 0.2 and 1 mM of isopropyl β-D-thiogalactopyranoside (IPTG), temperatures of 16 and 37°C, and shaking for 4 and 16 h. The control setup included bacterial cells that had not been induced. The bacterial cells were centrifuged at 12,000×*g* for 5 min, and the precipitate was resuspended with a buffer (20 mM Tris and 300 mM NaCl, pH 8.0), following which the recombinant MePMEI1 proteins were detected by sodium dodecyl sulfate polyacrylamide gel electrophoresis (SDS-PAGE). **Purification of MePMEI1 protein:** The expression of the recombinant MePMEI1 protein was induced under optimal induction conditions. The bacterial cells were subsequently collected by centrifugation at 12,000×*g* for 5 min, following which the precipitate was resuspended with a buffer (20 mM Tris and 300 mM NaCl, pH 8.0). The cells were sonicated on ice by ultrasonic disruption (Φ 3, 15%, 2 s/8 s, 10 min). The lysis solution was centrifuged at 12,000×*g* for 15 min at 4°C, and the recombinant MePMEI1 proteins in the supernatant and precipitate were detected by SDS-PAGE. The recombinant MePMEI1 proteins obtained from the precipitate solution were purified using a 2 mL Ni-Sepharose column. SDS-PAGE was used for analyzing the purified recombinant MePMEI1 protein. **Western blotting:** The protein separated by SDS-PAGE was electro-transferred to a polyvinylidene fluoride (PVDF) membrane. After washing three times with PBST (phosphate buffered saline, tween-20), the membrane was blocked with 5% skim milk powder in PBST for 2 h at room temperature. The recombinant MePMEI1 protein was verified using anti-His mouse monoclonal antibodies followed by staining with goat anti-mouse IgG in 5% skim milk powder in PBST for 1 h. The membrane was subsequently visualized with a Western Bright ECL kit, and the signal was detected using a chemiluminescence imaging system. Following renaturation, the purified recombinant MePMEI1 protein was used for analyzing the enzyme activity.

### Enzyme activity assay of recombinant protein

The assay used for determining the activity of PMEI was performed according to the method described by Francis et al. (2006) and Sun (2017), with slight modifications. Briefly, 0.5 μg of MePMEI1 protein was mixed with 0.8 μL of commercial orange peel PME (Sigma p5400) and added to 800 μL of pectin solution [0.1% esterified pectin (Sigma P9561) in 50 mM phosphate buffered saline (PBS), pH 7.0]. The MePMEI1 protein was not added to the control setup. The samples were incubated overnight at 37°C, following which 200 μL of 0.05% ruthenium red solution (Sigma R2751) was added and mixed, and the mixture was incubated for 15 min at room temperature. The samples were added to 200 μL of 0.6 M CaCl_2_ for precipitation, and the precipitate was collected by centrifugation at 12,000×*g* for 10 min. The absorbance of the supernatant was measured at A_534_. The changes in the activity of MePMEI1 were further determined at different pH (4.5, 5.5, 6.5, 7.5, and 8.5) and different temperatures (16, 25, 37, and 95°C). Three replicates were performed for each sample.

### Generation of transgenic Arabidopsis and plant growth

The Col-0 ecotype of *A. thaliana* was used in this study, and the floral dip method was used for transforming the plants. After vernalization at 4°C for 3 days, the seeds of Col-0 Arabidopsis were uniformly sown on vermiculite for germination, and the seedlings were transplanted singly to new vermiculite and cultured at 22°C under 16 h/8 h light/dark conditions (white LED, 120–150 uM/m^2^/S) till the time of flowering. The mature pods and fully opened flowers were cut off, and the Arabidopsis plants were used for transformation. *A. tumefaciens* containing the pCAMBIA1300-35s-*MePMEI1*-GFP recombinant vector was activated by streaking the plate, and the obtained single colony was cultured in YEP liquid medium at 28°C with shaking at 220 rpm until the OD_600_ reached 0.8–1.0. The bacterial cells were collected by centrifugation at 4,000 rpm for 10 min and resuspended by adding 1/2 MS liquid medium containing 0.03% L77 to an OD_600_ of 0.8. The flower buds of Arabidopsis were soaked in the resuspended bacterial liquid for 5 min, following which the plants were cultured in the dark for 24 h and subsequently grown at 22°C under 16 h/8 h light/dark conditions (white LED, 120–150 uM/m^2^/S). The infection was repeated every 2–3 days, for a total of 3–5 times, depending on the number of flowers. The infected Arabidopsis plants were cultured at 22°C under 16 h/8 h light/dark conditions (white LED, 120–150 uM/m^2^/S) to obtain the T0 seeds.

The T0 seeds of Arabidopsis were sterilized three times with 75% ethanol, washed with sterile water, and finally washed with absolute ethanol. Following vernalization at 4°C for 3 days, the seeds were placed on filter papers for drying and were subsequently germinated on 1/2 MS agar medium containing 25 mg/L hygromycin for screening the positive transgenic plants (T1). All the steps were performed under sterile conditions. The transgenic T1 Arabidopsis plants were identified by PCR using the 2 × M5 HiPer Superluminal mix-with blue dye (Mei5bio, Beijing, China). The DNA obtained from the transgenic plants was used as the template, and the specific primers used for PCR detection are enlisted in Supplementary Table 1. Three transgenic Arabidopsis plants with the highest *MePMEI1* expression were selected from the transgenic T1 plants by RT-qPCR for subsequent functional verification. The cDNA of the transgenic T1 Arabidopsis plants was used as the template. *Actin* was considered as the reference gene. The specific primers used for RT-qPCR are enlisted in Supplementary Table 1. The seeds of the three transgenic Arabidopsis plants were further screened on 1/2 MS agar medium containing 25 mg/L hygromycin until the homozygous T2 plants were obtained.

### Pb(II) treatments and evaluation of Pb resistance in Arabidopsis

The seeds of the transgenic homozygous T2 plants and WT Arabidopsis were sterilized as previously described and germinated on 1/2 MS agar medium following vernalization at 4°C for 3 days in the dark. The plants were grown in the culture room at 22°C under 16 h/8 h light/dark conditions (white LED, 120–150 uM/m^2^/S) for ~7 days. For each line, seedlings with similar growth status were transferred to 1/2 MS agar medium obtained 750 μM/1 mM Pb(NO_3_)_2_ or no-Pb(II) (control) and cultured for an additional 14 days. The three Arabidopsis plants from each line were horizontally positioned for observation and comparison.

The transgenic *MePMEI1* and WT Arabidopsis seedlings were collected for determining the accumulation of H_2_O_2_ and O^2−^ by diaminobenzidine (DAB) and nitroblue tetrazolium (NBT) staining following treatment with 750 μM Pb(NO_3_)_2_ for 14 days. NBT powder and a DAB Chromogenic Reagent were purchased from Beijing Solarbio Technology Co., Ltd. NBT and DAB were separately prepared to obtain solutions with concentrations of 0.05%. The samples of Arabidopsis seedlings were immersed in the solution for 20–30 min at room temperature under illumination. The staining solution was discarded, and the samples were washed three times with distilled water for terminating the reaction, following which 75% ethanol was added to the centrifuge tube, and the samples were boiled in water for 15 min until the chlorophyll had faded. Following decolorization, the samples were placed under a stereomicroscope for observing the color reaction.

### Transmission electron microscopy (TEM)

The structures of the cell wall in the roots and leaves of the transgenic and WT Arabidopsis under Pb stress or control conditions were observed by TEM. The plants were developed on 1/2 MS agar medium containing 750 μM Pb(NO_3_)_2_ or no-Pb(II) for 14 days, following which the roots and leaves of the seedlings were collected and the ultrathin sections were prepared. The sections were prepared as follows: the samples were fixed with 2% glutaraldehyde and 1% osmic acid, dehydrated with 30–50–70–80–90–100% ethanol and acetone, transferred to virgin resin for embedding and sectioning, and the sections were finally stained with uranyl acetate and lead citrate. The ultrathin sections thus prepared were observed under a transmission electron microscope (Hitachi).

### Determination of physiological and biochemical indices

The seeds of transgenic *MePMEI1* and WT Arabidopsis plants were uniformly sown on vermiculite for germination following vernalization at 4°C. When the plants had grown four true leaves, they were singly transplanted to vermiculite and grown under 16 h/8 h light/dark conditions (white LED, 120–150 uM/m^2^/S) at 22°C for 25 days. All the plants were irrigated with 5 mM Pb(NO_3_)_2_ solution every 2 days for ~16 days, while the control plants were irrigated with the same volume of tap water at the same frequency. The changes in the phenotype are continuously monitored during treatment. The activities of catalase (CAT) and superoxide dismutase (SOD) and the contents of malondialdehyde (MDA) and H_2_O_2_ were measured using the appropriate kits from Suzhou Comin Biotechnology Co., Ltd., according to the instructions provided. Three replicates were performed for each sample.

### Determination of PME activity in Arabidopsis

Following vernalization at 4°C for 3 days, the seeds of the transgenic plants with *MePMEI1* and the WT Arabidopsis plants were uniformly sown on vermiculite for germination, following which the seedlings were transplanted singly to new vermiculite and cultured under 16 h/8 h light/dark conditions (white LED, 120–150 uM/m^2^/S) at 22°C for 35 days. The activity of PME was determined using a Pectinesterase (PE) kit from Suzhou Comin Biotechnology Co., Ltd., according to the instructions provided. Three replicates were performed for each sample.

### Statistical analysis

Unless otherwise stated, all the numerical values were presented as the mean ± standard error of mean (SEM) of at least three independent biological replicates. The data were analyzed by a one-way analysis of variance (ANOVA) using SPSS software (version 22.0; SPSS Inc., Chicago, IL, USA). The different letters indicate significantly different means (*p* < 0.05, Duncan's multiple range tests). Microsoft Excel 2019 was used for data collation. The figures were prepared using GraphPad Prism software (GraphPad Prism 8.0.2, San Diego, CA, USA). The relative expression of the genes was calculated using the 2^−ΔΔCt^ method.

## Results

### MePMEI1 contains the conserved PMEI domain

In our previous study, we identified the *MePMEI1* gene with a coding region of 609 bp that encodes a protein of 202 amino acids. The estimated molecular weight and isoelectric point (pI) of the protein were determined to be 21.78 kDa and 5.51, respectively (Zhou et al., 2019).

**Figure 1:**
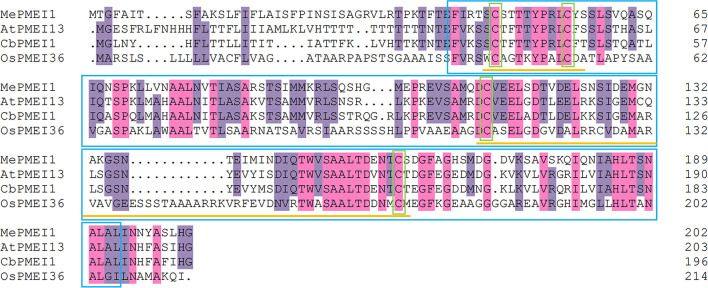
Multiple sequence alignment and analysis of conserved domains of the MePMEI1 protein and other PMEIs from different plant species. The purple and pink highlights indicate similar and identical residues, respectively. The PMEI domain and conserved cysteine residues are outlined in blue and green boxes, respectively. The disulfide bridges are underlined in yellow.

The AtPMEI13 protein of *A. thaliana* and the CbPMEI1 protein of *C. bungeana* are more homologous to the MePMEI1 protein of *M. esculenta* than with the OsPMEI36 protein of *O. sativa*, which is possibly attributed to the higher divergence between dicotyledons and monocotyledons. Multiple sequence alignment revealed that the MePMEI1 protein has a typical PMEI domain consisting of four conserved cysteine residues that formed two disulfide bridges (Figure 1).

### Pb(II) induces *MePMEI1* expression in cassava

**Figure 2:**
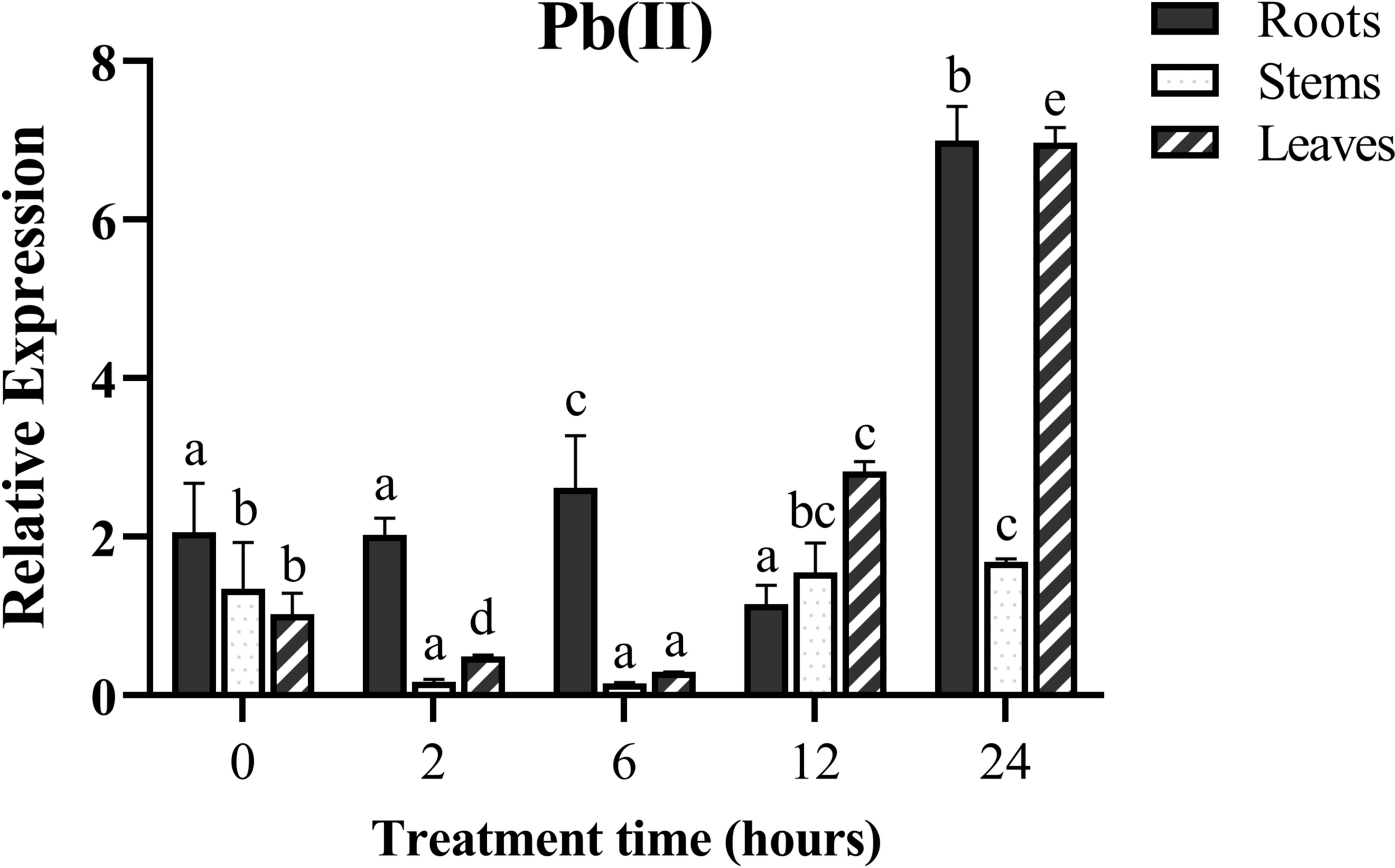
Expression of *MePMEI1* under Pb stress. The 45-days-old SC8 cassava seedlings were treated with 1 mM Pb(NO_3_)_2_ for 0, 2, 6, 12, and 24 h, following which the root, stem, and leaf samples were collected for RT-qPCR analysis. *Tubulin* was used as the reference gene. The data are presented as the mean ± SEM of observations from three biological replicates. The significant differences in *MePMEI1* expression in the tissues were analyzed at different time points. The different letters indicate significant differences at the 0.05 level (Duncan test).

To investigate whether the *MePMEI1* gene responds to Pb stress, the samples of the roots, stems, and leaves from the cassava plants treated with Pb(NO_3_)_2_ were collected for analyses with RT-qPCR. The transcription of *MePMEI1* was highest in the roots following treatment with 1 mM Pb(NO_3_)_2_, was slightly upregulated at first and then downregulated, and was again upregulated and finally reached a peak after 24 h of treatment. The transcription of *MePMEI1* was first downregulated in the stems and leaves, then upregulated, and reached a peak after 24 h of treatment (Figure 2). These results indicated that the *MePMEI1* gene of cassava responds to Pb stress, especially in the roots.

### MePMEI1 is localized in the cell wall

**Figure 3:**
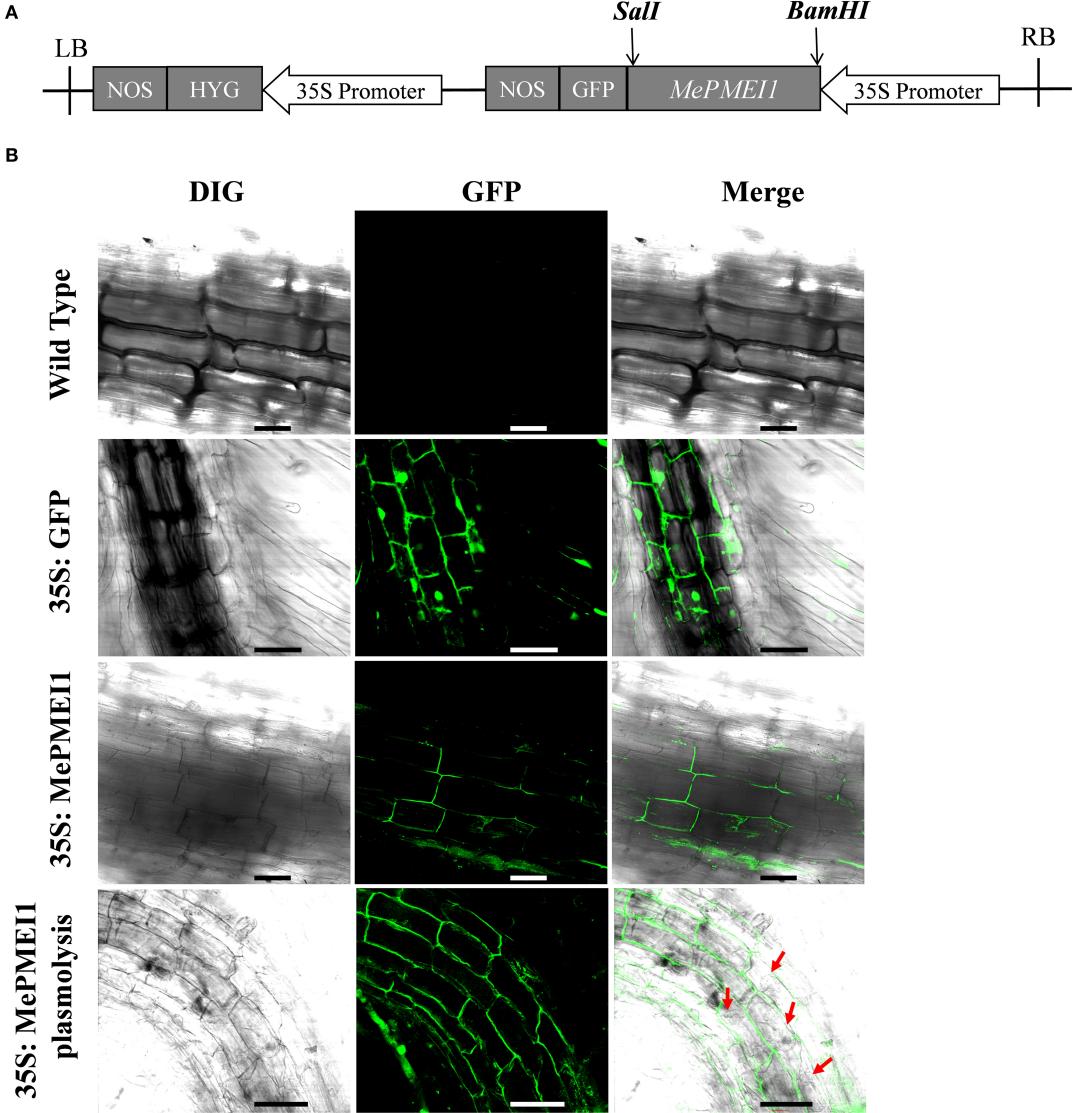
Analysis of the subcellular localization of MePMEI1. **(A)** A schematic diagram of the pCAMBIA1300-35s-*MePMEI1*-GFP recombinant vector. **(B)** Analysis of the localization of the MePMEI1 protein in the root tip cells of transgenic tobacco plants by observing the fluorescence signals. The red arrows indicate plasmolyzed cells. Scale bar = 60 μm.

To assess the subcellular localization of the MePMEI1 protein, the cells in the root tips of tobacco plants transformed with the pCAMBIA1300-35s-*MePMEI1*-GFP recombinant vector were observed under a laser confocal microscope (Figure 3A). Laser confocal microscopy revealed the absence of fluorescence signals in the WT tobacco plants. In the transgenic tobacco plants containing the pCAMBIA1300-35s-GFP vector, the fluorescence signal was distributed on the cell wall, the cell membrane, the intercellular spaces, and the nuclei. In the transgenic tobacco plants with *MePMEI1*, the fluorescence signal was observed in the cell wall, the cell membrane, and the intercellular layer. Following plasmolysis with 30% sucrose solution, the fluorescence signal was only observed on the cell wall, but not on the cell membrane, which exhibited a wrinkled morphology. The findings revealed that the MePMEI1 protein was therefore localized in the cell wall (Figure 3B).

### *MePMEI1* inhibits activity of commercial PME

**Figure 4:**
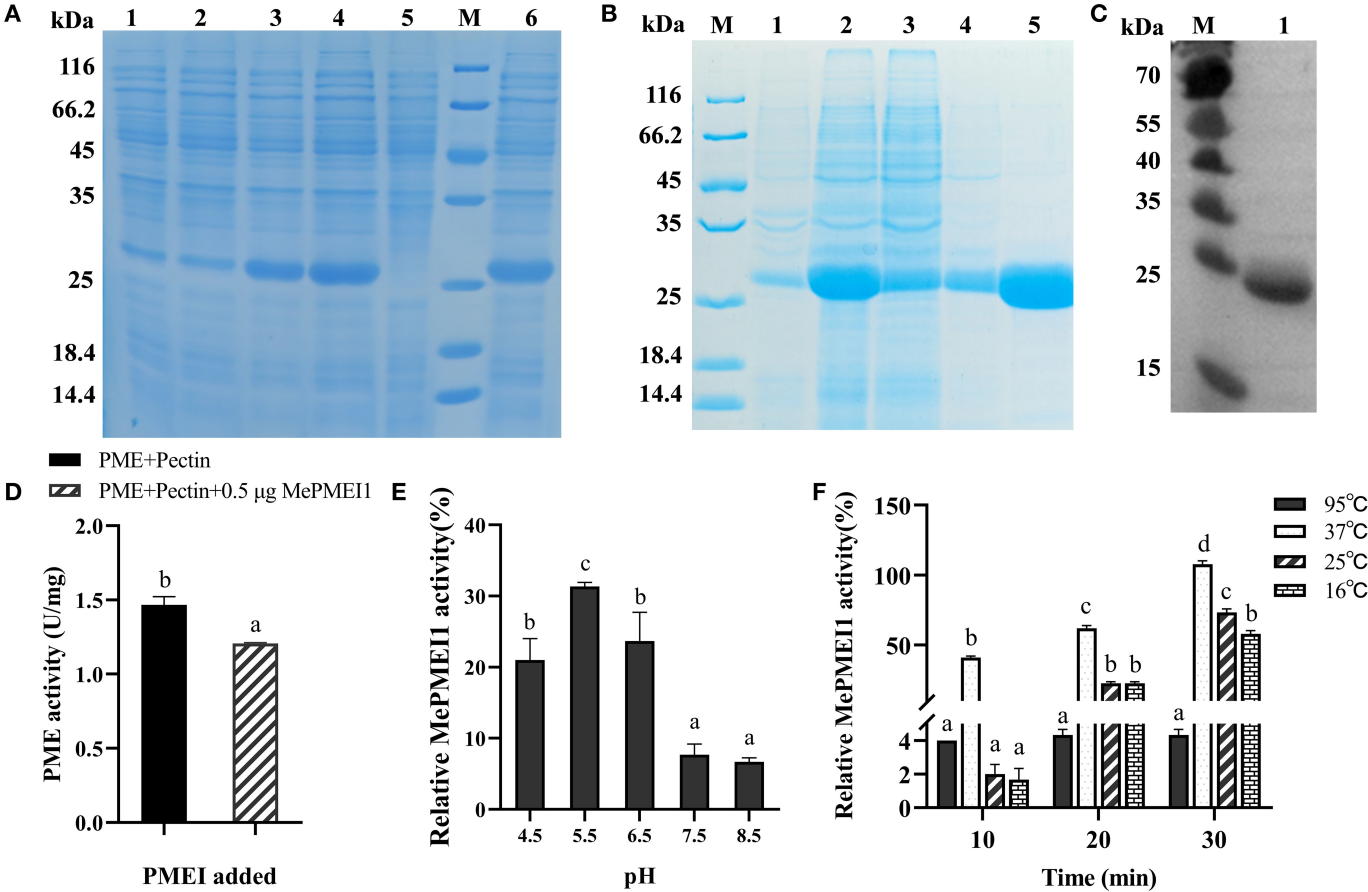
Determination of the expression and biological functions of MePMEI1. **(A)** Expression of the crude MePMEI1 recombinant protein under different conditions of induction; lane 1: 15°C, 0.2 mM IPTG; lane 2: 15°C, 1 mM IPTG; lane 3: 37°C, 0.2 mM IPTG; lane 4: 37°C, 1 mM IPTG; lane 5: non-induced cells; and lane 6: insoluble protein fraction after induction with 1 mM IPTG at 37°C. **(B)** Expression of purified recombinant MePMEI1 protein in the sediment and supernatant following the disruption of inclusion bodies (lanes 1 and 2), protein effluent (lane 3), and flushed out a sample (lane 4). Lane 5 depicts the expression of the purified recombinant MePMEI1 protein. **(C)** Western blotting for the detection of the recombinant MePMEI1 protein; lane 1: recombinant MePMEI1 protein; M: molecular marker (kDa). **(D)** Assay for determining PME activity following the addition of the recombinant MePMEI1 protein. Effects of different **(E)** pH and **(F)** temperature on the activity of MePMEI1. The data are presented as the mean ± SEM of observations from triplicate assays. The different letters indicate significant differences at the 0.05 level (Duncan test).

The BL21 Star (DE3) *E. coli* cells containing the PET-30a (+)-*MePMEI1* recombinant vector carrying His tags were induced with different concentrations of IPTG (0.2 mM and 1 mM) at different temperatures (15 and 37°C). A specific band for MePMEI1 with a molecular mass of 27 kDa was observed under all induction conditions (Figure 4A, lanes 1–4). It was observed that the expression of the recombinant MePMEI1 protein was higher at 37°C when induced with 1 mM IPTG (Figure 4A, lane 4). Solubility analysis revealed that the induced protein was expressed as an inclusion body (Figure 4A, lane 6). A large proportion of the insoluble protein was purified and examined by SDS-PAGE (Figure 4B). The results of western blotting using an anti-his antibody revealed a specific band at ~27 kDa, which was consistent with the expected results (Figure 4C). The results indicated that the recombinant MePMEI1 protein was successfully expressed in the prokaryotic expression system.

The effect of the recombinant MePMEI1 protein on PME activity was determined by analyzing the enzyme activity. The initial activity value of commercial orange peel PME was 1.47 U/mg at room temperature, which was reduced to 1.2 U/mg following the addition of 0.5 μg of recombinant MePMEI1 protein, indicating that MePMEI1 had an obvious inhibitory effect on commercial orange peel PME (Figure 4D). To explore the effects of pH on the activity of MePMEI1, the recombinant MePMEI1 protein and commercial orange peel PME were incubated with a pectin solution at different pH (4.5, 5.5, 6.5, 7.5, and 8.5). The relative activity of MePMEI1 was represented by subtracting the PME activity value at different pHs when incubated with the recombinant MePMEI1 protein, from the initial activity value of PME at the corresponding pH. We observed that the highest MePMEI1 activity of 31% was observed at pH 5.5 and then decreased with increasing pH. The results demonstrated that the activity of MePMEI1 is higher under acidic conditions, and the optimum pH was determined to be 5.5 (Figure 4E). We also determined the activity of MePMEI1 at different temperatures. The recombinant MePMEI1 protein and commercial orange peel PME were incubated with a pectin solution at 16, 25, 37, and 95°C. The experimental data revealed that the recombinant MePMEI1 protein showed the strongest PME inhibition at 37, 25, and 16°C, and the difference in the activity of recombinant MePMEI1 at 37°C was statistically significant. The activity of MePMEI1 increased with the duration of incubation; however, the protein was inactivated and had no inhibitory effect on PME at 95°C (Figure 4F).

### Transgenic *MePMEI1* Arabidopsis exhibited enhanced Pb tolerance and altered cell wall properties

To evaluate the role of *MePMEI1* in Arabidopsis, we obtained five Arabidopsis plants with transgenic *MePMEI1* by agrobacterium-mediated transformation using the floral dip method. Three transgenic *MePMEI1* lines (OE#2, OE#4, and OE#5 from T3) with higher *MePMEI1* transcription levels were selected by RT-qPCR for subsequent verification of function (Supplementary Figure 1).

**Figure 5:**
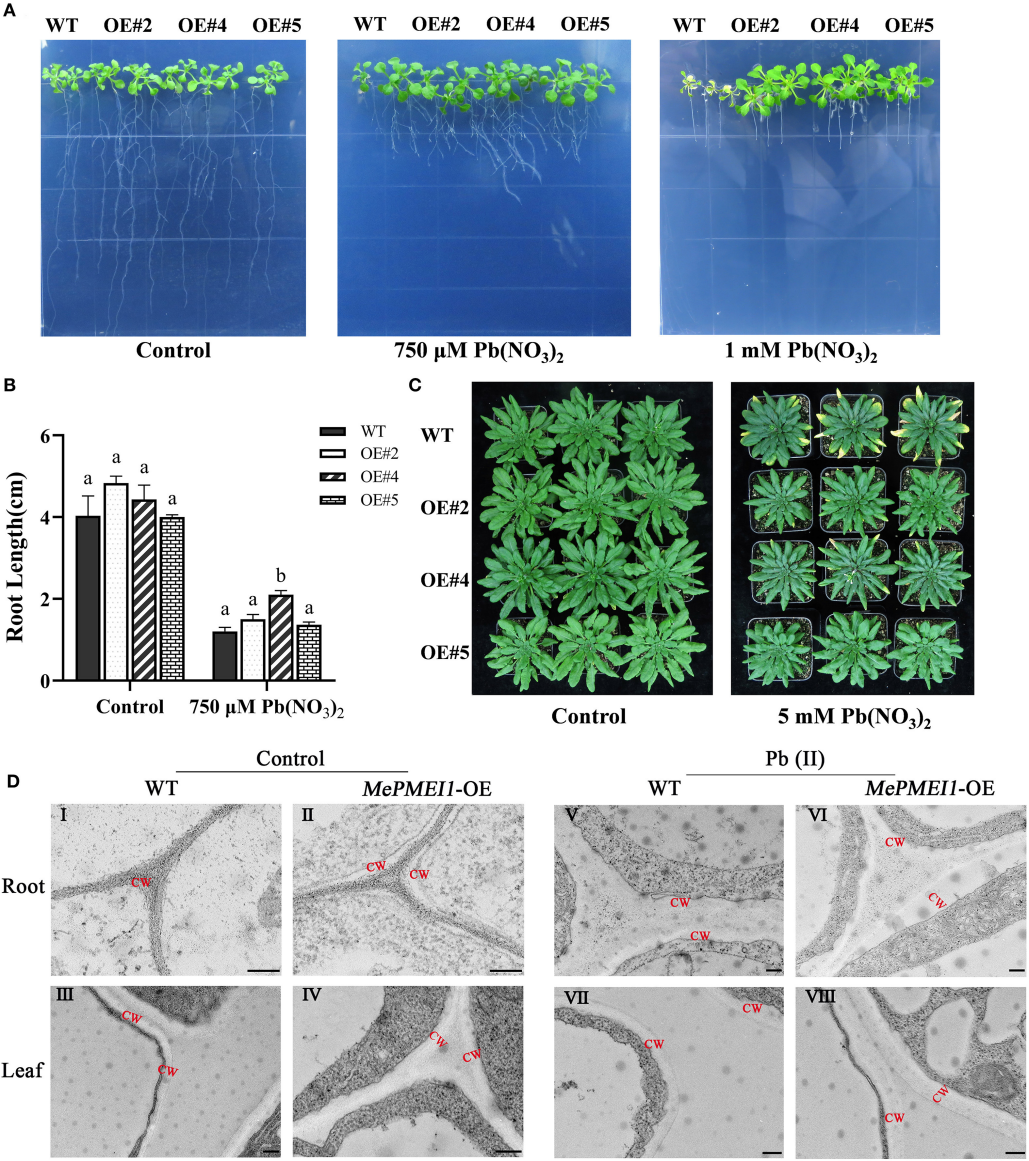
Resistance assay of Arabidopsis under Pb stress. The growth of the transgenic *MePMEI1* and WT plants under control conditions or following treatment with Pb(NO_3_)_2_ when grown on **(A)** 1/2 MS agar medium for 14 days or **(C)** vermiculite for 16 days. **(B)** Statistical analysis of the length of plant roots. The data are presented as the mean ± SEM of observations from three biological replicates. The different letters indicate significant differences at the 0.05 level (Duncan test). **(D)** The structure of the cell walls in root tips (I, II, V, and VI) and leaves (III, IV, VII, and VIII) of transgenic *MePMEI1* and WT Arabidopsis under control conditions (I–IV) and following treatment with 750 μM Pb(NO_3_)_2_ (V–VIII) for 14 days as observed by TEM. CW: cell wall. Scale bars = 200 nm.

Root elongation was markedly inhibited in all 7-days-old Arabidopsis seedlings following treatment with 750 μM or 1 mM Pb(NO_3_)_2_ for 14 days; however, the above-ground parts of Arabidopsis seedlings with transgenic *MePMEI1* were larger than those of WT plants, and root growth was also significantly increased in the transgenic plants compared to that of the WT plants. In particular, the WT seedlings failed to grow and tended to die following treatment with 1 mM Pb(NO_3_)_2_, while the transgenic *MePMEI1* plants exhibited increased growth characteristics (Figures 5A,B). One the vermiculite, the 25-days-old transgenic *MePMEI1* plants and the WT plants were treated with tap water or 5 mM Pb(NO_3_)_2_ solution for 16 days. The results demonstrated that the above-ground parts of all the plants remained green and the plants grew well under normal conditions. The edges of the rosette leaves of WT plants turned yellow following treatment with 5 mM Pb(NO_3_)_2_; however, the rosette leaves of transgenic *MePMEI1* plants remained green (Figure 5C). The results indicated that *MePMEI1* promotes tolerance to Pb stress. We also evaluated the survival rate of seeds and found that treatment with Pb(NO_3_)_2_ clearly affected the rooting of Arabidopsis seedlings, but there was no significant difference in rooting between transgenic *MePMEI1* plants and WT plants, under normal conditions and Pb stress (Supplementary Figure 2).

The structures of the cell wall in the root tips and leaves of transgenic and WT plants under normal conditions and under 750 μM Pb(NO_3_)_2_ treatment were observed by TEM. The images revealed that the cell wall in the root tips and the leaves of transgenic *MePMEI1* plants were significantly thicker than those of WT plants under normal conditions (red letters indicate the cell wall) in Figure 5DI–IV. The positive effect of *MePMEI1* on the thickening of cell walls was more obvious following treatment with 750 μM Pb(NO_3_)_2_ for 14 days. The cell walls appeared thicker in both transgenic and WT plants following treatment; however, the cell walls in the root tips and the leaves of seedlings with transgenic *MePMEI1* were much thicker than those of the WT plants (Figure 5DV–VIII). The results demonstrated that the overexpression of *MePMEI1* increased the thickness of the cell wall in Arabidopsis.

### *MePMEI1* improved excessive ROS scavenging under Pb stress in transgenic plants

**Figure 6:**
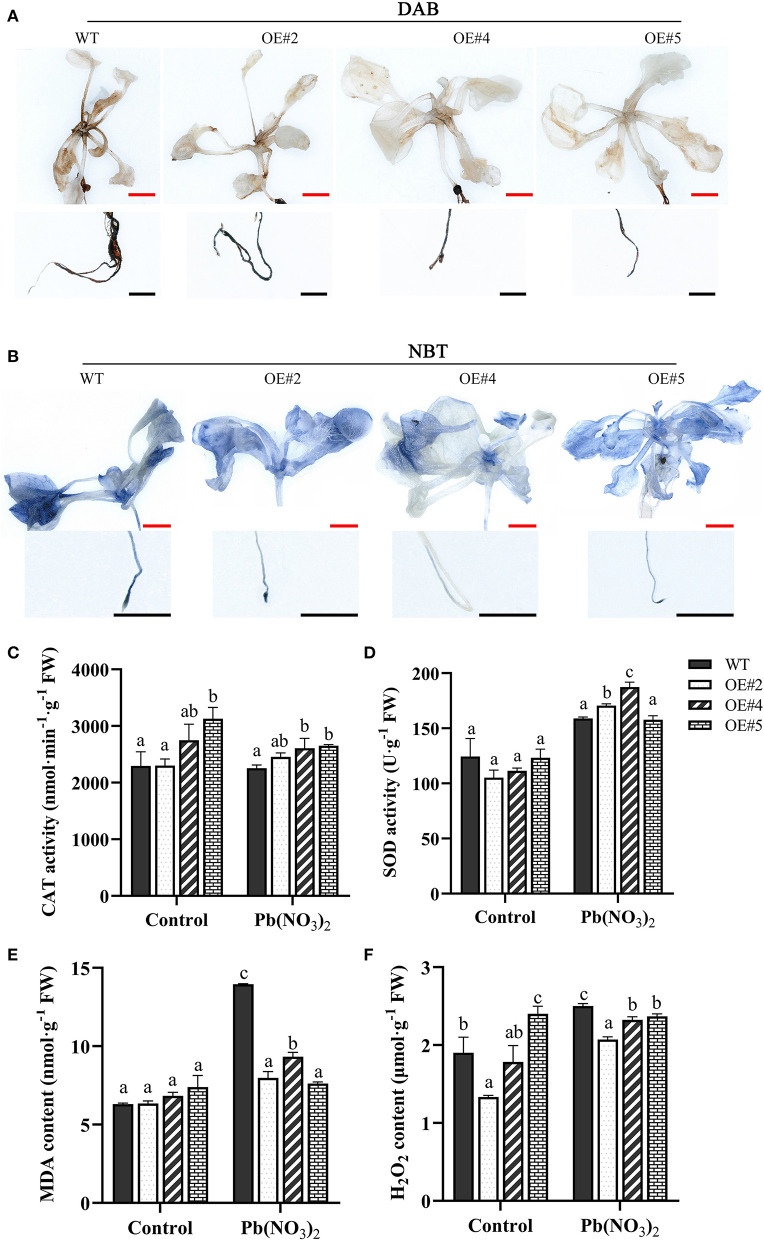
Analysis of ROS accumulation and the activities of the antioxidant enzymes. Accumulation of H_2_O_2_ and O^2−^ in transgenic *MePMEI1* and WT Arabidopsis seedlings by **(A)** DAB and **(B)** NBT staining following treatment with 750 μM Pb(NO_3_)_2_ for 14 days. The images were captured using a stereomicroscope. Black scale bars = 500 μm, red scale bars = 1 mm. Activities of **(C)** CAT and **(D)** SOD, and the contents of **(E)** MDA and **(F)** H_2_O_2_ in transgenic *MePMEI1* and WT plants under control conditions and following treatment with Pb(NO_3_)_2_. The data are presented as the mean ± SEM of observations from three biological replicates. The different letters indicate significant differences at the 0.05 level (Duncan test).

Several studies reported that plants usually produce higher quantities of ROS under heavy metal stress. To investigate whether *MePMEI1* confers ROS scavenging potential, the accumulation of H_2_O_2_ and O^2−^ in the root tips and leaves of transgenic *MePMEI1* and WT plants following treatment with 750 μM Pb(NO_3_)_2_ for 14 days was determined by DAB and NBT staining. A higher quantity of brown precipitate was detected in the root tips and leaves of WT plants following DAB staining than in those of the transgenic *MePMEI1* lines (Figure 6A), indicating that the accumulation of H_2_O_2_ was lower in the transgenic plants than in the WT plants. The quantity of O^2−^ was additionally examined by NBT staining. The results demonstrated a higher quantity of blue precipitate in the root tips and leaves of WT Arabidopsis plants than in those of the transgenic *MePMEI1* lines (Figure 6B), indicating that the expression of *MePMEI1* reduced the accumulation of O^2−^ in Arabidopsis under Pb stress.

The content of MDA and H_2_O_2_ and the activity of the antioxidant enzymes, CAT and SOD, were further measured in the transgenic and the WT plants under control conditions and following treatment with 5 mM Pb(NO_3_)_2_ for 16 days. The results demonstrated that the activities of CAT and SOD were higher in all the transgenic *MePMEI1* lines than in the WT plants, while the levels of MDA and H_2_O_2_ were higher in the WT plants than in the transgenic plants under Pb treatment (Figures 6C–F). These findings illustrated that *MePMEI1* alleviated oxidative damages under Pb stress by enhancing the activities of CAT and SOD for reducing the excess accumulation of ROS.

To determine the effect of *MePMEI1* on the activity of PME in Arabidopsis, we also examined the total PME activity in 35-days-old Arabidopsis plants. The results demonstrated that the PME activity was reduced in transgenic Arabidopsis compared to that of WT plants (Supplementary Figure 3). These findings were consistent with the earlier results and indicated that the *MePMEI1* functions as a PMEI and inhibits the activity of plant PMEs.

## Discussion

Plant cell walls act as the first barrier against xenobiotic attacks; therefore, maintaining the integrity of cell walls is critical for stress resistance in plants. Numerous studies demonstrated that PMEIs interact with PMEs to regulate the degree of pectin methylesterification and affect the firmness of cell walls (Wormit and Usadel, 2018), thereby regulating numerous biological processes related to plant growth (Pelletier et al., 2010; Wu et al., 2017; Wormit and Usadel, 2018; Lu et al., 2020). Additionally, it has been demonstrated that PMEIs partake in plant resistance to environmental stress (Lionetti et al., 2007, 2017; An et al., 2008; Nguyen et al., 2016; Chen et al., 2018; Liu et al., 2018). However, the response of PMEIs to heavy metals remains to be investigated. This study investigated the role of the *MePMEI1* gene in modulating plant resistance to Pb exposure *via* the regulation of plant cell walls.

Consistent with the existing reports on stress resistance, we observed that treatment with Pb(II) induced the expression of *MePMEI1* in the roots, stems, and leaves of cassava plants, as analyzed by RT-qPCR. The expression of *MePMEI1* was relatively higher in the roots than in the other plant parts, which was probably attributed to the fact that the roots are the first to respond to heavy metals and are the first binding area for metal ions (Gupta et al., 2013) (Figure 2). Subsequent analyses of subcellular localization revealed that MePMEI1 was localized in the cell walls (Figure 3B). *MePMEI1* was found to be homologous to *AtPMEI13* and *CbPMEI1*, which have been demonstrated to participate in cold and salt stress. The proteins encoded by *MePMEI1, AtPMEI13*, and *CbPMEI1* were found to contain the typical PMEI domain (Figure 1). Enzymatic analysis revealed that MePMEI1 inhibited commercial PME, which implied that MePMEI1 functions as a PMEI in cassava (Figure 4D). Altogether, these results clearly demonstrated that the functions of the protein encoded by *MePMEI1* may be similar to those of PMEIs involved in diverse stress responses, including plant response to drought, salt, cold, and heat stress, and indicated that the tolerance of cassava plants to Pb stress is mediated *via* MePMEI1 functioning in the cell walls.

It has been reported that Pb toxicity negatively affects plant growth by reducing plant height, inducing leaf wilting and chlorosis, reducing the content of soluble proteins, decreasing the flowering rate, and suppressing root development (Gupta et al., 2011; Zhang et al., 2020). Pb exposure also induces a burst in the production of ROS, including H_2_O_2_, O^2−^, and OH^−^, and increases the accumulation of MDA, which is accompanied by an increase in the activities of antioxidant enzymes, including CAT, SOD, and ascorbic acid (AsA) (Gupta et al., 2011; Huang et al., 2012). The excess production of ROS can damage plant cells. In this study, the transgenic Arabidopsis plants with *MePMEI1* showed high tolerance to Pb stress (Figures 5A–C). The activities of CAT and SOD were higher in the transgenic *MePMEI1* Arabidopsis plants under Pb stress compared to those of WT plants. The enhanced antioxidant activity could therefore alleviate the accumulation of ROS for promoting plant tolerance to Pb stress (Figure 6). This could account for the enhanced growth of transgenic *MePMEI1* plants compared to WT plants under Pb stress. Altogether, these results clearly illustrated that the antioxidant enzymes, CAT and SOD, enhanced plant tolerance to heavy metal (Pb) stress.

Pb is one of the most serious pollutants in the soil and is extremely harmful to organisms, impairing food safety and public health (Singh et al., 2013). Phytoremediation is a safe and inexpensive biotechnique for overcoming the negative consequences and remediating Pb pollution in soils, as metal ions are thought to accumulate in the cell walls of roots (Sun and Luo, 2018). Previous studies demonstrated that the absorption and accumulation of Cd are higher in the roots than in the above-ground parts of low-Cd variety plants, which confirmed that the cell walls of roots are the binding sites for Cd^2+^ and also act as a barrier for preventing the entry of ions into the cytoplasm (Wang et al., 2020; Ma et al., 2021). The plants used for phytoremediation usually have strong tolerance and can transport or adsorb contaminants under environmental stress (Gupta et al., 2013; Wu et al., 2013). It has been demonstrated that numerous metal transporters, such as those encoded by *AtPDR8* and *AtPSE1*, are involved in the transportation of metal ions, and their expression is positively correlated with plant growth under metal ion stress (Kim et al., 2007; Fan et al., 2016). The present study demonstrated that the *MePMEI1* gene can also increase plant tolerance to Pb stress by inducing the thickening of plant cell walls. This led us to speculate that a large number of negatively charged groups in pectin can absorb Pb ions, which show a higher accumulation in cell walls and are prevented from entering the cytoplasm, thus reducing damage to plants (Loix et al., 2017; Spain et al., 2021). Numerous studies demonstrated that pectin can directly bind metal ions, including Cd^2+^, Al^3+^, and Cu^2+^, and conversely affect the content and structure of pectin (Wang et al., 2020). Altogether, we believe that such PMEIs can serve as candidate molecules during phytoremediation for inhibiting the entry of Pb into intracellular organelles. However, to achieve this, quite a few issues need to be clarified through further research.

We subsequently determined that the inhibitory effect of MePMEI1 on PME activity was strongest at pH 5.5 (Figure 4E). Similar results have been reported in a study on kiwifruits, which reported that the structure of PMEIs is loosened beyond a pH of 6, accompanied by the breakage of intra-protein disulfide bonds, which eventually weakens the ability of PMEIs to bind and inhibit PMEs (Bonavita et al., 2016). The secondary structures of PMEIs are irreversibly altered beyond a pH of 7.5, resulting in the disruption of S–S bridges, indicating a reduction in the degree of PMEI-PME interactions (Wormit and Usadel, 2018). It can therefore be speculated that different PMEIs have different effects at different pH. These results demonstrated that the function of MePMEI1 can be enhanced by adjusting the pH of the environment.

In conclusion, the present study elucidates the molecular basis of the *MePMEI1* gene, which encodes the PMEI protein in cassava, in increasing plant tolerance to Pb stress. Overall, this study highlights the key role of *MePMEI1* in driving plant tolerance to Pb toxicity by regulating the structure of plant cell walls. These findings can aid in the development of *MePMEI1*-mediated plant breeding techniques for enhancing heavy metal tolerance and also provide useful insights for controlling Pb-contaminated soils *via* phytoremediation measures in the future.

## Data availability statement

The original contributions presented in the study are included in the article/Supplementary materials, further inquiries can be directed to the corresponding authors.

## Author contributions

YY, XH, and JG guided the experiments, revised the manuscript, and were mainly responsible for project administration and fund acquisition. YZ performed the experiment and prepared the manuscript. RL designed and supervised the research. SW performed data collection and analysis. YW, JL, ZD, and QZ performed the experiments. All authors contributed to the study and approved the final manuscript.

## Funding

This research was supported by the National Key R&D Program of China (2019YFD1001105 and 2018YFD1000500), the National Natural Science Foundation of China (31601359), the Earmarked Fund for China Agriculture Research System (CARS-11-HNGJC), The Major Science and Technology plan of Hainan Province (ZDKJ2021012), and the Central Public-interest Scientific Institution Basal Research Fund for Chinese Academy of Tropical Agricultural Sciences (1630052022008).

## Conflict of interest

The authors declare that the research was conducted in the absence of any commercial or financial relationships that could be construed as a potential conflict of interest.

## Publisher's note

All claims expressed in this article are solely those of the authors and do not necessarily represent those of their affiliated organizations, or those of the publisher, the editors and the reviewers. Any product that may be evaluated in this article, or claim that may be made by its manufacturer, is not guaranteed or endorsed by the publisher.
