# Supplementary material for: Overexpression of MePMEI1 in Arabidopsis enhances Pb tolerance
Source: Front Plant Sci. 2022 Sep 16;13:996981. doi: 10.3389/fpls.2022.996981 (PMC9523724; doi:10.3389/fpls.2022.996981)
Supplement: Supplementary file 1 [file Data_Sheet_1.docx]

**Supplementary Material**

Table S1. Primers used in experiments

| Name | Upstream primer (5’-3’) | Downstream primer (5’-3’) |
| --- | --- | --- |
| pCAMBIA1300-MePMEI1 | AGCGTCGACATGACGGGTTTTGCCATTA | CGCGGATCCACCATGAAGAGAGGCATA |
| PET-30a (+)-MePMEI1 | GTCCATATGGGTAGAGTTCTCAGAACCC | CTCCTCGAGACCATGAAGAGAGGCATAA |
| pCAMBIA1300-DNA | TTTGGAGAGAACACGGGGGA | CAGGGTCAGCTTGCCGTAG |
| MePMEI1-Qpcr-At | GAAATGGGCAATGCTAAAGG | CAGGTATTCTCATCCGTCAA |
| MePMEI1-Qpcr-Me | CGGTCCAAGCAAGCCAGAT | ACTCAACTCCTCCACGCAG |
| Tubulin | GTGGAGGAACTGGTTCTGGA | GTGGAGGAACTGGTTCTGGA |
| Actin | CGTTTGTGGGAATGGAAGCT | TTGCTCATACGGTCAGCGATA |


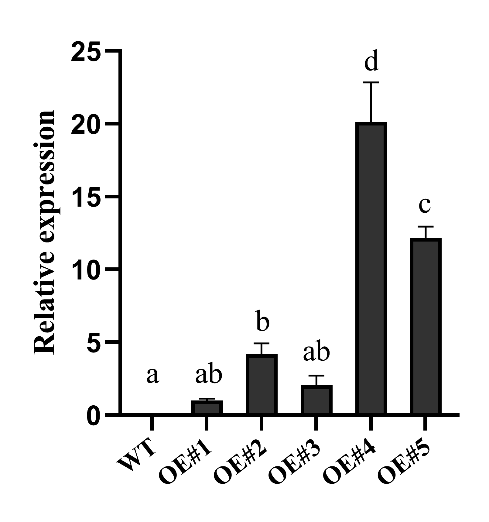


**Figure S1** Transcription level detection of *MePMEI1* gene in transgenic and WT Arabidopsis by RT-qPCR. The data are presented as the mean ± SEM of observations from three biological replicates. The different letters indicate significant differences at the 0.05 level (Duncan test).

**Figure S2** The seeds survival rate of transgenic *MePMEI1* and WT Arabidopsis under control conditions or following treatment with 750 μM Pb (NO_3_)_2_.


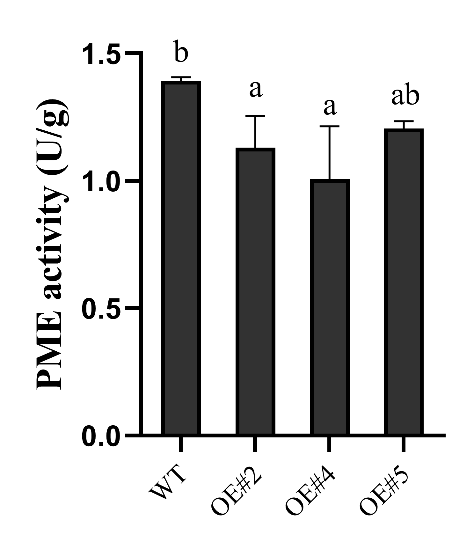


**Figure S3** The PME activity examination in Arabidopsis. The data are presented as the mean ± SEM of observations from three biological replicates. The different letters indicate significant differences at the 0.05 level (Duncan test).
